# Prediction and suppression of internal blue discoloration in roots of daikon, the Japanese radish (*Raphanus sativus* L.)

**DOI:** 10.1002/fsn3.774

**Published:** 2018-09-17

**Authors:** Katsunori Teranishi, Masayasu Nagata

**Affiliations:** ^1^ Graduate School of Bioresources Mie University Tsu Mie Japan; ^2^ Food Research Institute National Agriculture and Food Research Organization Tsukuba Ibaraki Japan

**Keywords:** 4‐hydroxyglucobrassicin, daikon, hydrogen peroxide, internal blue discoloration, Japanese radish, oxidative stress

## Abstract

The internal blue discoloration of edible daikon roots often occurs on day 3 after harvest during storage at 20°C and is a serious problem. This study reports a rapid and simple method for predicting discoloration at harvest and proposes two methods for suppressing the discoloration of roots that are at discoloration risk. The soaking of freshly harvested roots in aqueous hydrogen peroxide resulted in immediate blue discoloration. The correlation between discoloration after storage at 20°C and discoloration after soaking in hydrogen peroxide was positive. Discoloration using hydrogen peroxide at harvest is a useful way of predicting discoloration risk. The storage of roots at 10°C in air or at 20°C in an atmosphere containing 1% (v/v) molecular oxygen resulted in no discoloration for at least 8 days. These storage conditions can guarantee no discoloration for the distribution after harvest.

## 1 INTRODUCTION

**Figure 1:**
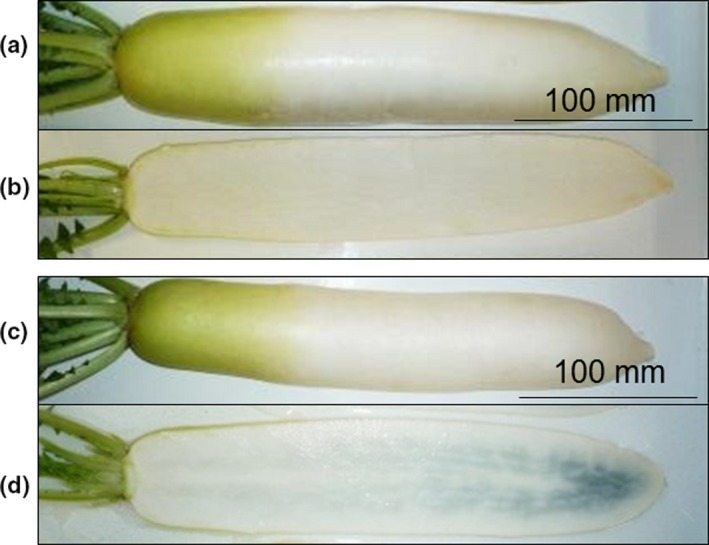
Appearance of the (a) outside and (b) inside of freshly harvested daikon root and the (c) outside and (d) inside of daikon root after storage at 20°C for 4 days

Daikon, the Japanese radish (*Raphanus sativus* L.), is an important, abundantly produced agricultural crop in Japan and is consumed as an edible white root vegetable (Figure 1 a,b). In Japan, daikon is cultivated on approximately 32,300 hectares, with the production of approximately 1.36 million tons in 2016 (Data from a portal site for Japanese government statistics, http://www.maff.go.jp/j/tokei/kouhyou/sakumotu/index.html). To improve the appearance, harvesting efficiency, disease resistance, and taste of daikon roots, daikon breeding is in progress. Over the past decade in Japan, the appearance of an internal blue color has been observed in white daikon roots during distribution following harvest (Figure 1 c,d) (Ikeshita, Ishibata, & Kanamori, 2010). This physiological phenomenon does not occur at the time of harvest, as shown in Figure 1b, and is only observed by consumers during preparation for cooking, leading to complaints to shopkeepers and farmers. Although the internal blue discoloration has not been reported to be harmful, the commodity value of affected daikon is reduced because of the strange blue color in the white roots. Thus, this is a serious issue for both shopkeepers and farmers.

**Figure 2:**
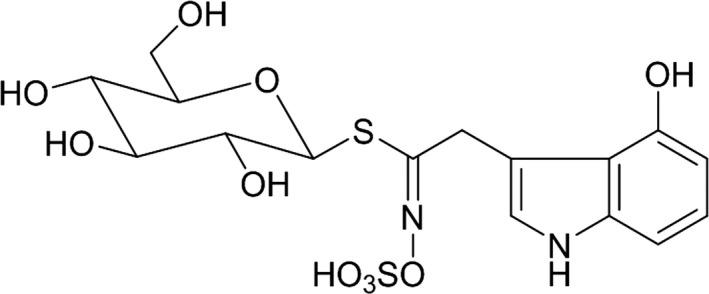
Chemical structure of 4‐hydroxyglucobrassicin

Another discoloration phenomenon, internal browning in white daikon roots, has been studied by Japanese researchers since the 1980s. This discoloration is induced by high soil temperatures during the root maturation period (Fukuoka & Enomoto, 2001; Fukuoka & Kanamori, 1990). The occurrence of brown discoloration depends on sowing date, cultivar, and fertilizer conditions (Kawai, Hikawa, & Fujiwara, 1992; Kawai, Hikawa, & Ono, 1993; Kawashiro & Takeda, 1983) and correlates with the accumulation of polyphenolic compounds in the internal regions of the roots (Kawai et al., 1992). Regarding the internal blue discoloration, in 2016 we showed that the only precursor for the formation of blue‐colored compounds is 4‐hydroxyglucobrassicin (Figure 2); blue components are formed by oxidation of the precursor molecule with reactive oxygen species produced from hydrogen peroxide in the presence of peroxidase (Teranishi & Nagata, 2016). Generation of the blue color is therefore a result of oxidative stress within daikon roots, leading to the oxidation of 4‐hydroxyglucobrassicin and the production of blue‐colored molecules (Teranishi, Nagata, & Masuda, 2016). 4‐Hydroxyglucobrassicin also works as an antioxidative compound, akin to ascorbic acid, and the blue discoloration is a real‐time indicator of extreme oxidative stress in the roots. Although 4‐hydroxyglucobrassicin, which was first discovered in cabbage seed and rapeseed in 1982 (Truscott, Burke, & Minchinton, 1982), is generally contained in the seeds, sprouts, and roots of *Brassica* plants (Agerbirk & Olsen, 2012), this was the first observation that 4‐hydroxyglucobrassicin generates blue‐colored compounds in plants. 4‐Hydroxyglucobrassicin has been known to be unstable and is converted to a blue‐colored compound(s) (Sang & Truscott, 1984). The oxidation mechanism was investigated using 4‐hydroxyindole as a model, which indicated that oxidation proceeds via the formation of indol‐(*4,7*)‐*p*‐quinone (Latxague & Gardrat, 1992; Truscott & Manthey, 1989).

The aim of the current study was to develop simple and rapid methods for predicting internal blue discoloration in daikon roots at the time of harvest and methods to suppress discoloration during storage based on the molecular mechanism underlying the blue discoloration. If the prediction of blue discoloration indicates a high discoloration risk, the daikon lots need to be stored under suitable conditions to avoid the discoloration. The likelihood of the blue discoloration depends on the cultivar of daikon and the conditions of cultivation (Ikeshita et al., 2010). Thus, a method to predict the risk of discoloration for each lot and discoloration‐suppressing storage conditions for each lot is required.

## 2 MATERIALS AND METHODS

### 2.1 Plant materials and cultivation

A total of 29 cultivars of daikon were used in the current study. *Raphanus sativus* L. cv. Hukuhomare*, Hukuhomare**, Hukutenka*, and MKS‐828* daikon seeds were purchased from Mikado Kyowa Seed Co., Ltd., Chiba, Japan; *R. sativus* L. cv. KJ‐505*, NR‐515**, NR‐516**, and Yuuto** daikon seeds were from Nanto Seed Co., Ltd., Nara, Japan; *R. sativus* L. cv. SC9‐149** and SC8‐260* daikon seeds were from Sakata Seed Co., Ltd., Kanagawa, Japan; *R. sativus* L. cv. MRX‐012**, MRX‐119**, MRX‐120*, and YR‐2* daikon seeds were from Marutane Co., Ltd., Kyoto, Japan; *R. sativus* L. cv. Aodumari‐2*, Aodaisyou‐2*, Natsumidori‐5**, Natsumidori‐8**, and Yosaku* daikon seeds were from Nakahara Seed Co., Ltd., Hukuoka, Japan; *R. sativus* L. cv. 11‐4050* and Akitouge* daikon seeds were from Tohoku Seed Co., Ltd., Tochigi, Japan; *R. sativus* L. cv. Gensuke**, Hakusyu**, Taibyousoubutori*, and TDA‐732* daikon seeds were from Takii Seed Co., Ltd., Kyoto, Japan; *R. sativus* L. cv. Natsuza** daikon seeds were from Yamato Noen Co., Ltd., Nara, Japan; *R. sativus* L. cv. Huyunourasoubutori** daikon seeds were from Kaneko Seeds Co., Ltd., Gunma, Japan; *R. sativus* L. cv. TRI‐9918* daikon seeds were from Tokita Seed Co., Ltd., Saitama, Japan; *R. sativus* L. cv. IN‐BR1104* daikon seeds were from Watanabe Seed Co., Ltd., Saitama, Japan; *R. sativus* L. cv. Sorotane* daikon seeds were from Sinzienta Seed Co., Ltd., Tokyo, Japan; and *R. sativus* L. cv. RA‐305* daikon seeds were from Yukizirusi Seed Co., Ltd., Hokkaido, Japan. The daikon cultivars appended with * and ** were grown in an open field at the Kanagawa Agricultural Center in 2012 and in an open field at the Ishikawa Sand Dune Agricultural Research Center in 2012, respectively, using conventional cultivation methods. Hukuhomare was cultivated in 2017 also by farmers in Kanazawa using conventional cultivation methods.

### 2.2 Chemicals

Chemicals were purchased from Wako Pure Chemical Industries Ltd., Osaka, Japan. The concentration of hydrogen peroxide was determined using the standard titration with KI and Na_2_S_2_O_3_.

### 2.3 Storage experiments

**Figure 3:**
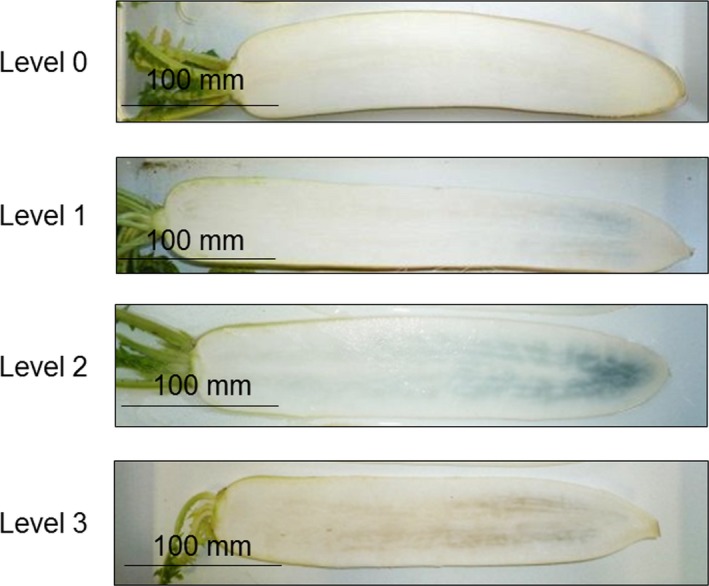
Index for the three levels 0–3 of discoloration after storage of freshly harvested daikon roots at 20°C and approximately 80% relative humidity for 4 days in the dark. The blue discoloration eventually changes to brown (level 3)

Freshly harvested roots of 29 cultivars of daikon (*n* = 5) were washed with water and stored at 20°C and approximately 80% relative humidity for 4 days in open air and in the dark using the MLR‐350HT growth cabinet (Sanyo Electric Co., Ltd., Osaka, Japan). The roots were vertically cut along the middle, and the degree of discoloration of the root sections was visually assessed for the intensity, but not the area, of blue discoloration and was expressed as four levels from 0 to 3 in order of increasing discoloration as shown in Figure 3. The results are expressed as the mean of the samples from five roots.

For the temperature storage study, freshly harvested roots of Hukuhomare daikon cultivated in 2017 were washed with water and stored at 10, 12.5, 15, 17.5, or 20°C at approximately 80% relative humidity in the dark in MLR‐350HT growth cabinet, and the roots were vertically cut along the middle on day 2, 3, 4, 6, or 8. The degree of discoloration was visually assessed in the manner described above.

For the oxygen tension study, freshly harvested roots of Hukuhomare daikon cultivated in 2017 were washed with water and stored at 20°C and approximately 80% relative humidity in two different gas mixtures, O_2_:CO_2_:N_2_ = 1:1:98 and 5:1:94 (v), using a WKN‐9200EX O_2_/CO_2_ incubator (Waken B Tech, Kyoto, Japan). Alternatively, roots were stored at 20°C in soil containing 12% (w/w) water. The roots were vertically cut along the middle on day 2, 3, 4, 6, or 8, and the degree of discoloration was visually assessed in the manner described above.

### 2.4 Blue discoloration using hydrogen peroxide

**Figure 4:**
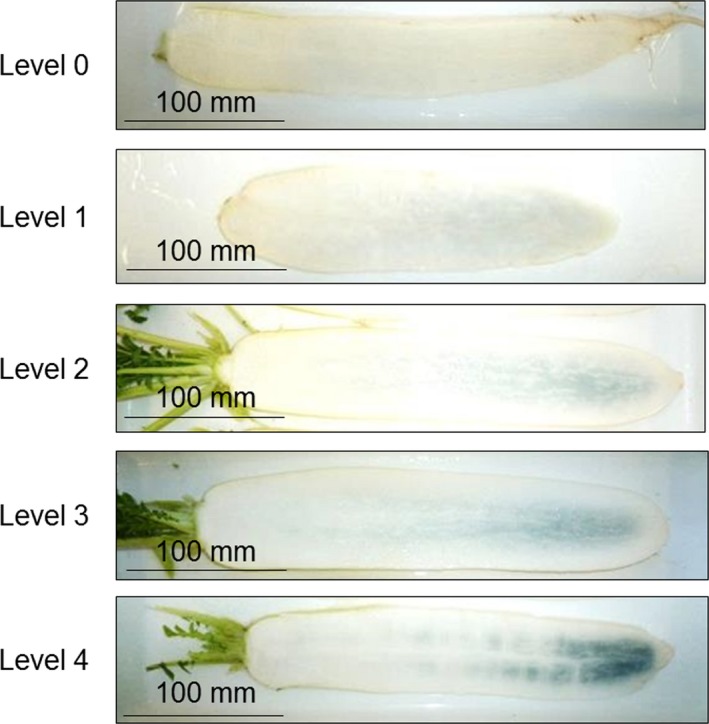
Index for the levels 0–4 of discoloration after treatment of freshly harvested daikon roots with 1% aqueous hydrogen peroxide at 20°C for 10 min

Freshly harvested roots of 29 cultivars of daikon (*n* = 5) were washed with water, vertically cut along the middle, and soaked in 1% (w/w) aqueous hydrogen peroxide solution at 20°C. The intensity (but not the area) of the resulting color change was visually assessed after 10 min of soaking the roots in 1% aqueous hydrogen peroxide solution, and each sample was assigned a value from 0 to 4 in order of increasing discoloration, as shown in Figure 4. The results are expressed as the mean value of samples from five roots.

#### 2.4.1 Hydroxyglucobrassicin analysis

Xylem sections (5.0 g) at 50 mm from the root tip of Hukuhomare stored at 10°C in air, at 20°C in air, at 20°C in the gas mixture, O_2_:CO_2_:N_2_ = 1:1:98 (v), and at 20°C in soil containing 12% (w/w) water were immediately immersed in methanol (5 ml) and frozen at −80°C, and the mixtures were homogenized in an ice bath. The homogenates were filtered through absorbent cotton. The resulting filtrates were centrifuged at 10,000 g at 0°C for 10 min, and the supernatants were filtered through 0.45‐μm membrane filters (Millex‐HP; Merck Millipore Ltd., Carrigtwohill, Co. Cork, Ireland). The resultant filtrates were stored at −80°C until further use. 4‐Hydroxyglucobrassicin in the filtrates was analyzed using a JASCO Gulliver HPLC system equipped with a photodiode array (PDA) detector MD‐910 (JASCO Corp., Tokyo, Japan), and a Cosmosil 5C18‐PAQ column (4.6 mm × 250 mm) Nacalai Tesque, Inc., Kyoto, Japan) at 20°C. The mobile phase was a mixture of aqueous 0.1% (v/v) trifluoroacetic acid solution (A) and 0.1% (v/v) trifluoroacetic acid/MeOH solution (B). The flow rate was 0.8 ml/min in a linear gradient starting with 0% B and reaching 50% B in 20 min. The injection volume of sample was 20 μl. 4‐Hydroxyglucobrassicin was identified using the PDA detector with monitoring between 210 and 650 nm and, subsequently, using a ZQ 4000 MS spectrometer (Waters Corporation, Milford, MA) in the electrospray negative ionization mode. MS parameters were as follows: cone voltage, 30 V; source temperature, 120°C; desolvation temperature, 350°C; gas flow (N_2_), 350 L/hr. The concentration of 4‐hydroxyglucobrassicin in the extracts was quantified on the basis of absorbance at 280 nm using calibration curves of chemically synthesized 4‐hydroxyglucobrassicin (Teranishi & Nagata, 2016). The content in the sections is reported as the mean ± *SD* of samples from five roots and is expressed as nanomoles per gram of wet weight.

## 3 RESULTS AND DISCUSSION

The Hukuhomare daikon cultivar has been known to suffer from internal blue discoloration in Japan. We investigated the degree of the discoloration in 29 daikon cultivar roots, including Hukuhomare, on the 4th day of storage at 20°C. The resulting blue color intensities were scored on a visual analogue scale as shown in Figure 3, and the scores are reported in Table 1. Hukuhomare, RA‐305, NR‐515, MRX‐120, and Aozumari‐2 scored the highest for internal blue discoloration. The degree of discoloration of both Hukuhomare daikon cultivated at the Kanagawa Agricultural Center and at the Ishikawa Sand Dune Agricultural Research Center was the same. Taibyousoubutori, Yuuto, Gensuke, Hakusyu, and Huyunourasoubutori daikon produced no discoloration. The internal blue discoloration has been known to turn brown with time in the same region of the root (Teranishi & Nagata, 2016). We present an example to brown discoloration in Figure 3, although no cultivars developed brown discoloration after 4 days in this study.

**Table 1:** Degree of discoloration after 1% aqueous hydrogen peroxide treatment or after storage at 20°C for 4 days

Cultivar	Degree of blue discoloration with hydrogen peroxide	Degree of internal discoloration after storage
Taibyousoubutori*	0 ± 0	0 ± 0
Yuuto**	0.4 ± 0.55	0 ± 0
Gensuke**	1 ± 0	0 ± 0
Hakusyu**	1 ± 0	0 ± 0
Huyunourasoubutori**	0.8 ± 0.45	0 ± 0
11‐4050*	2 ± 0	1 ± 0
Aodaisyou‐2*	2 ± 0	1.2 ± 0.45
IN‐BR1104*	2 ± 0	0.8 ± 0.45
MKS‐828*	2 ± 0	0.8 ± 0.45
MRX‐119**	2.4 ± 0.55	0.6 ± 0.55
Natsuza**	1.8 ± 0.45	0.8 ± 0.45
NR‐516**	2 ± 0	1 ± 0
TDA‐732*	2 ± 0	1 ± 0
TRI‐9918*	2 ± 0	1 ± 0
Sorotane*	2 ± 0	0.4 ± 0.55
Yosaku*	2 ± 0	0.8 ± 0.45
Natsumidori‐5**	3 ± 0	1.8 ± 0.55
SC9‐149**	2.6 ± 0.55	1.8 ± 0.55
Akitouge*	3 ± 0	1 ± 0
SC8‐260*	2.8 ± 0.45	0.4 ± 0.55
Aozumari‐2*	3.4 ± 0.55	2 ± 0
Hukutenka*	3.4 ± 0.55	1.8 ± 0.45
KJ‐505*	3.2 ± 0.45	1.8 ± 0.45
MRX‐012**	3 ± 0	1.8 ± 0.45
MRX‐120*	3 ± 0	2 ± 0
Natsumidori‐8**	3 ± 0	1.6 ± 0.55
NR‐515**	3.5 ± 0.55	2 ± 0
YR‐2*	2.8 ± 0.45	1.8 ± 0.45
RA‐305*	3.2 ± 0.4	2 ± 0.48
Hukuhomare*	4 ± 0.45	2 ± 0
Hukuhomare**	3.8 ± 0.45	2 *±* 0

Notes

Data presented are mean values ± *SD* of five roots.

Cultivars marked with * and ** were cultivated at the Kanagawa Agricultural Center and at the Ishikawa Sand Dune Agricultural Research Center, respectively.

Recently, we discovered that soaking fresh Hukuhomare daikon root sections in aqueous hydrogen peroxide solution induces artificial blue discoloration (Teranishi & Nagata, 2016). In the current study, the artificial blue discoloration of Hukuhomare daikon root sections increased with the increase in soaking time in 0.01, 0.1, or 1% aqueous hydrogen peroxide solution at room temperature. The degree of the discoloration reached saturation within 5–30 min. Soaking in a 1% solution produced a high degree discoloration that can be readily distinguished by visual assessment, whereas at 0.01%, the color change is too low for reliable detection (Supporting information Figure S1). Thus, in this study, soaking in 1% aqueous hydrogen peroxide solution at 20°C for 10 min was employed for the visual assessment. Samples of the same 29 fresh cultivars of daikon were treated with 1% hydrogen peroxide for comparison with the internal blue discoloration produced after storage at 20°C for 4 days. The visual evaluation of blue discoloration on the 29 cultivars, using the color index in Figure 4, is summarized in Table 1. The Hukuhomare cultivar produced the highest degree of blue discoloration, and Taibyousoubutori produced the lowest. Yuuto, Gensuke, Hakusyu, and Huyunourasoubutori daikon, which produced no internal blue discoloration during storage at 20°C for 4 days, generated only slight blue discoloration after treatment with 1% hydrogen peroxide.

**Figure 5:**
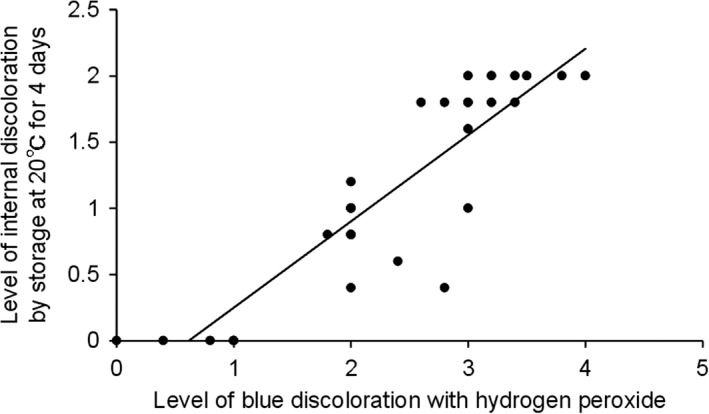
Relationship between levels of blue discoloration after treatment with 1% hydrogen peroxide and of internal discoloration after storage at 20°C for 4 days. Data presented are mean values ± *SD* of samples from five roots

The relationship between discoloration by treatment with 1% aqueous hydrogen peroxide and in storage at 20°C for 4 days is shown in Figure 5. There was a positive correlation between the two datasets (*R* ^2^ = 0.79), indicating that discoloration with hydrogen peroxide facilitates a visual prediction of internal blue discoloration. If the degree of blue discoloration after hydrogen peroxide treatment of freshly harvested roots was above 2, then internal blue discoloration was likely to occur upon storage at 20°C, and a farmer would be advised to store the roots under conditions that suppress the discoloration. This prediction method using 1% aqueous hydrogen peroxide would be convenient for farmers to use for visual assessment because of the low concentration of hydrogen peroxide required, the minimal costs involved, and the simple and rapid operation of the method that requires no technical expertise or analytical equipment.

In Japan, edible daikon roots are sold at supermarkets within a week of harvest. Thus, when the prediction test for internal blue discoloration using hydrogen peroxide by sampling inspection at the time of harvest indicates a high risk of discolouration, suitable measures must be taken to prevent discolouration in those daikon lots for at least 1 week.

**Figure 6:**
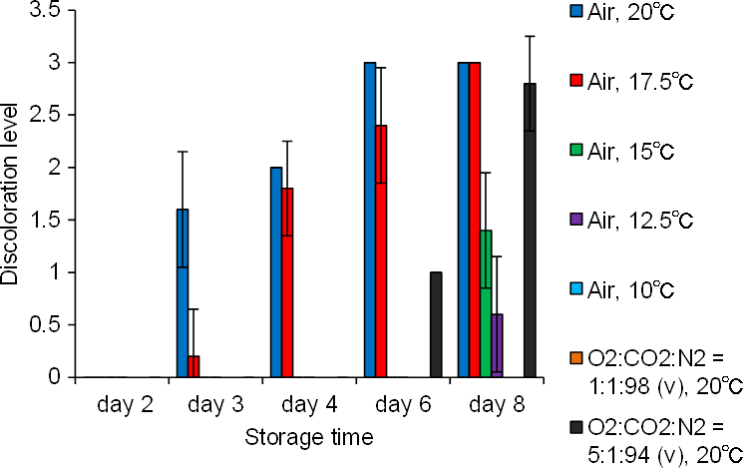
Effects of temperature and molecular oxygen concentration at the time of storage on internal discoloration. Data presented are mean values ± *SD* of samples from five roots. When stored at 10°C in air or at 20°C in the mixed gas O_2_:CO_2_:N_2_ = 1:1:98 (v), no internal blue discoloration occurred for 8 days

**Figure 7:**
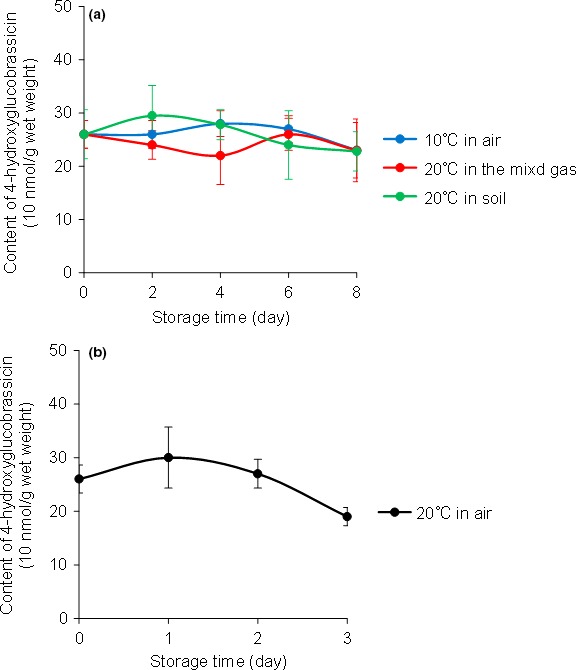
Contents of 4‐hydroxyglucobrassicin in xylem sections obtained at 50 mm from the root tip of Hukuhomare daikon stored at (a) 10°C in air, at 20°C in a mixed gas of O_2_:CO_2_:N_2_ = 1:1:98 (v), at 20°C in soil, or at (b) 20°C in air. Data are presented as the mean values ± *SD* of samples from five roots. When stored at 20°C in air, internal blue discoloration occurred on day 3, and the 4‐hydroxyglucobrassicin content was not measured after that

The internal blue discolouration occurs by chemical oxidation of 4‐hydroxyglucobrassicin under the oxidative stress in daikon roots (Teranishi et al., 2016). We speculated that oxidative stress could be suppressed by lowering the storage temperature. The cost of temperature control, however, should be as low as possible to maintain economic viability. We therefore sought to determine the highest storage temperature that would permit storage of roots for 1 week with no discolouration. For this purpose, we used Hukuhomare daikon, which produced the highest degree of internal blue discolouration during storage at 20°C. We stored roots for up to 8 days at five different temperatures and determined internal discolouration levels at different times (Figure 6). No discolouration occurred after 8 days of storage at 10°C, whereas slight internal blue discolouration occurred on day 8 at 12.5°C. Thus, storage at 10°C is effective in suppressing the discolouration for a week while minimizing cost. The 4‐hydroxyglucobrassicin content, which was analyzed using an HPLC‐PDA‐MS system (Supporting information Figure S2), in the roots stored at 10°C did not change significantly (Figure 7a), and the 4‐hydroxyglucobrassicin content at 8 days of 10°C storage was similar to that at 2 days at 20°C, which was before the blue discoloration occurred (Figure 7b). When 4‐hydroxyglucobrassicin contents were similar, the blue discoloration from 4‐hydroxyglucobarssicin could be used as a real‐time indicator of extreme oxidative stress. Therefore, these results indicate that the suppression of blue discoloration at 10°C was not due to the 4‐hydroxyglucobrassicin content but because of the suppression of oxidative stress.

The effect of molecular oxygen concentration on the onset of blue discoloration was also investigated (Figure 6). Storage of Hukuhomare daikon at 20°C in the mixed gas O_2_:CO_2_:N_2_ = 1:1:98 (v) resulted in no discoloration for 8 days, whereas storage in O_2_:CO_2_:N_2_ = 5:1:94 (v) produced internal blue discoloration on day 6. The 4‐hydroxyglucobrassicin content in the roots did not significantly fluctuate for 8 days (Figure 7a). Thus, the lack of discoloration was due to the suppression of oxidative stress. Although 4‐hydroxyglucobrassisin gets immediately oxidized with hydrogen peroxide in the presence of peroxidase in a test tube (Teranishi & Nagata, 2016), oxidation by molecular oxygen under the same conditions is much slower. It has been suggested that low molecular oxygen concentration does not decrease 4‐hydroxyglucobrassicin oxidation directly but rather by suppressing the generation of reactive oxygen species formed during oxidative stress. When fresh Hukuhomare daikon roots were stored in soil at 20°C for 8 days, no internal blue discoloration occurred, and 4‐hydroxyglucobrassicin content did not change significantly (Figure 7a). The Hukuhomare daikon used in the current study were cultivated in open‐field soil and were harvested in October, when the average daily temperature was 17–22°C; internal blue discoloration was not observed at the time of harvest. The result of the storage experiment using soil, therefore, is consistent with that of cultivation in an open field. These results indicate that it is only when daikon roots are directly exposed to air (i.e., at harvest) that oxidative stress begins and the development of blue discoloration ultimately results.

## 4 CONCLUSIONS

The internal blue discoloration of daikon roots during storage at approximately 20°C after harvest has recently been recognized as a serious problem for farmers. Therefore, a method for predicting discoloration at the time of harvest and suitable storage measures for daikon lots at high discoloration risk is both needed. We found that artificial blue discoloration induced by soaking daikon sections in aqueous hydrogen peroxide solution can predict the risk of internal blue discoloration during storage at 20°C. This simple visual assessment method would be practical and useful, particularly when used in conjunction with the storage methods we developed. The storage of high‐risk daikon lots at 10°C produced no discoloration after 8 days, and lowering the concentration of molecular oxygen to 1% (v/v) also prevented discoloration onset for 8 days at 20°C. 4‐Hydroxyglucobrassicin content did not change significantly for 8 days under these storage conditions, indicating that oxidative stress was suppressed by these conditions. These storage methods can help prevent discoloration for a week after harvest.

## CONFLICT OF INTEREST

The authors declare that they do not have any conflict of interest.
