# Supplementary material for: Prediction and suppression of internal blue discoloration in roots of daikon, the Japanese radish (Raphanus sativus L.)
Source: Food Sci Nutr. 2018 Sep 17;6(8):2134–40. doi: 10.1002/fsn3.774 (PMC6261226; doi:10.1002/fsn3.774)
Supplement: Supplementary file 1 [file FSN3-6-2134-s001.docx]

Supporting information

Title: Prediction and suppression of internal blue discolouration in roots of daikon, the Japanese radish (*Raphanus sativus* L.)

Running title: Internal blue discoloration in daikon

Author: Katsunori Teranishi^1*^ and Masayasu Nagata^2^

^1^Graduate School of Bioresources, Mie University, 1577 Kurimamachiya, Tsu, Mie, Japan

^2^Food Research Institute, National Agriculture and Food Research Organization, 2-1-12 Kannondai, Tsukuba, Ibaraki, Japan

^*^Correspondence to: K. Teranishi, Graduate School of Bioresources, Mie University, 1577 Kurimamachiya, Tsu, Mie, Japan. E-mail address: teranisi@bio.mie-u.ac.jp.

**Figure S1.** Discoloration after treatment of freshly harvested Hukuhomare daikon roots with (A) 0.01 %, (B) 0.1 %, or (C) 1 % aqueous hydrogen peroxide at 20 °C for 10 min.

**Figure S2.** Representative HPLC chromatogram of extract obtained from Hukuhomare root. The arrow indicates the peak in chromatogram 4-hydroxyglucobrassicin. The extract was analyzed using a JASCO Gulliver HPLC system equipped with a PDA detector MD-910 (JASCO Corp., Tokyo, Japan), and a Cosmosil 5C18-PAQ column, Nacalai Tesque, Inc., Kyoto, Japan) at 20 °C. The mobile phase was a mixture of aqueous 0.1% (v/v) trifluoroacetic acid solution (A) and 0.1% (v/v) trifluoroacetic acid/MeOH solution (B). The flow rate was 0.8 mL/min in a linear gradient starting with 0% B and reaching 50% B in 20 min.
